# The influence of occupational stress and job satisfaction on burnout among healthcare workers in the UAE: A cross-sectional study

**DOI:** 10.1371/journal.pone.0335430

**Published:** 2025-10-28

**Authors:** Yousef Aljawarneh, Ahmad Al-Bashaireh, Zainab Albikawi, Osama Alkouri, Nour Ali Alrida, Mohammad Abuadas, Alanoud Alobaidly, Mahrah Alyileili, Shayma Alyileili, Wadha Alyileili, Fatmah Alhefetti, Amna Alhefeiti, Abdulkareem Alshehri, Abdulhafith Alharbi, Omar Qaladi

**Affiliations:** 1 Department of Nursing, Faculty of Health Sciences, Higher Colleges of Technology, Abu Dhabi, United Arab Emirates; 2 Faculty of Nursing, Philadelphia University, Amman, Jordan; 3 Faculty of Nursing, Yarmouk University, Irbid, Jordan; 4 Staff Development Unit, Nursing Department, Jaber Alahmed Armed Forces Hospital, Al-Wusta, Kuwait; 5 Department of Nursing, Faculty of Health Sciences, Higher Colleges of Technology, Abu Dhabi, United Arab Emirates; 6 Advanced Diagnostic and Therapeutic Institute, King Abdulaziz City for Science and Technology (KACST), Riyadh, Saudi Arabia; 7 College of Nursing, University of Hail, Hail, Saudi Arabia; 8 Community and Psychiatric Mental Health Nursing Department, King Saud University, Riyadh, Saudi Arabia; Endeavour College of Natural Health, AUSTRALIA

## Abstract

**Background:**

Healthcare Workers (HCWs) frequently face high levels of occupational stress, job dissatisfaction, and burnout due to the demanding nature of their work. In the United Arab Emirates (UAE), these challenges are further intensified by the rapid growth of the healthcare sector and increasing workloads, making it particularly critical to study these variables. Therefore, this study aimed to investigate the influence of occupational stress and job satisfaction on burnout and to identify the key predictors of burnout among HCWs in the UAE.

**Methods:**

A cross-sectional study was conducted among 498 HCWs from hospitals and primary healthcare centers under Emirates Health Services/ Ministry of Health and Prevention. Data was collected using the Work Stress Questionnaire (WSQ), Maslach Burnout Inventory (MBI), and Minnesota Satisfaction Questionnaire (MSQ).

**Results:**

The mean level of occupational stress was moderate, with a mean of 34.68 (SD = 10.15). The most affected subscales were “work-to-leisure time interference” and “influence at work.” The level of job satisfaction was also moderate, with a mean of 3.13 (SD = 0.75), and the highest satisfaction was related to extrinsic factors. The levels of burnout were notably high for emotional exhaustion and depersonalization, whereas “personal accomplishment” was less affected. Stepwise multiple regression analysis revealed significant predictors of emotional exhaustion (R^2^ = 0.530), including individual demands, work-to-leisure conflict, job satisfaction, income, and marital status. Depersonalization was predicted by indistinct organization, income, and employment type (R^2^ = 0.254). The least affected personal accomplishment subscale was predicted by occupational stress, age, education, nationality, and working hours, accounting for 6.9% of the variance (R^2^ = 0.069).

**Conclusion:**

The present study has highlighted the urgent need for targeted interventions to reduce occupational stress and improve job satisfaction to combat burnout among HCWs in the UAE. Organizational strategies should focus on workload management, promoting a healthy work-life balance, and clearly defining roles. These findings offer a foundation for informed policy actions to safeguard HCW well-being and elevate healthcare quality.

## Background

The healthcare environment is inherently complex and multifaceted, characterized by various stressors that significantly impact the well-being of Healthcare Workers (HCWs). These stressors include high patient loads, emotional demands, lack of resources, and pressure to ensure patient safety [1]. The impact of these stressors on HCWs is profound, as it influences not only individual well-being but also the quality of care provided to patients [1]. The UAE healthcare system’s workforce, including doctors, nurses, and allied health professionals, regularly confronts high workloads, shift rotations, emotional demands, and, at times, inadequate organizational support factors that are well-documented predictors of elevated stress and burnout, and diminished job satisfaction [2,3]. Occupational stress among HCWs is a significant concern that has garnered increasing attention in recent years. Defined as the harmful physical and emotional responses that occur when the demands of the job exceed the capabilities of the worker, occupational stress can lead to a range of adverse outcomes, including mental health issues, decreased job performance, and compromised patient safety [4]. International research has consistently highlighted that healthcare workers are at significant risk for occupational stress and burnout compared to many other professions. Studies conducted in the Middle East and North Africa have shown considerable prevalence rates, with up to 68% of healthcare workers reporting high stress levels as a result of shift work, role ambiguity, excessive workloads, and lack of administrative or collegial support [3,5,6]. Within the UAE, local studies have yielded similar findings, highlighting issues such as work-life imbalance, insufficient recognition, and organizational factors unique to the region [7,8]. The COVID-19 pandemic has further exacerbated these challenges, leading to increased anxiety, depression, and intentions to leave the profession [9]. This is consistent with findings from Saudi Arabia, where healthcare workers reported elevated levels of stress and burnout during the pandemic, driven by increased patient loads and emotional exhaustion [10]. Additionally, the COVID-19 pandemic exacerbated existing stressors, underscoring the need for comprehensive strategies that include social support and resilience training.

Job satisfaction, directly influenced by job demands, organizational support, interpersonal relationships, and opportunities for professional growth, plays a pivotal role in the retention and engagement of healthcare professionals [7,11]. Evidence from studies in the UAE indicates that recognition, fair career advancement policies, and supportive leadership are among the strongest predictors of healthcare staff retention and satisfaction [7,12]. The prevalence of job satisfaction among HCWs varies significantly in different regions and healthcare settings. For example, in a study conducted in Saudi Arabia, 39% of healthcare professionals reported high levels of job satisfaction, while only 6% showed strong dissatisfaction [13]. In contrast, studies from Nepal reported that 71.1% of the health workers were ambivalent about their job satisfaction during the COVID-19 pandemic.

Conversely, when these factors are lacking, the risk of burnout increases markedly [14,15]. The World Health Organization (WHO) describes burnout as a syndrome caused by chronic workplace stress that has not been effectively managed. It is characterized by emotional exhaustion, mental detachment or cynicism toward one’s job, and a decline in professional effectiveness. Notably, the WHO emphasizes that burnout is an occupational phenomenon, not a medical disorder [16].

Burnout is not an isolated psychological event; rather, it is often the result of the interplay between high occupational stress and low job satisfaction, leading to negative consequences for both healthcare workers and patient care outcomes [16–18]. In the UAE, cross-sectional and qualitative research during and after the COVID-19 pandemic has indicated that female healthcare workers, those with long working hours, and professionals in high-risk departments report the highest prevalence of burnout and dissatisfaction [2,9]. Similarly, studies from other regions reflect comparable patterns. For instance, 62% of health workers in acute care settings in Tanzania showed signs of burnout, particularly emotional exhaustion [19]. Likewise, in China, research among obstetricians and pediatricians found that 56.6% of participants experienced occupational burnout, with key contributing factors including poor doctor–patient relationships and high workload [20].

The Job Demands-Resources (JD-R) theory provides a well-established framework for understanding how workplace characteristics impact employees’ well-being, particularly in demanding sectors such as healthcare. According to the JD-R model, all occupations have specific risk factors related to job stress that can be classified as either job demands or job resources. “Job demands” are aspects of a job requiring sustained physical, emotional, or mental effort, such as high workload, shift work, emotional challenges, and ambiguity, which can lead to increased physiological and psychological costs (such as stress or burnout) if not balanced by sufficient resources [21–23]. “Job resources,” conversely, are aspects that help in achieving work goals, reduce job demands, or stimulate personal growth and development, including organizational support, autonomy, career development, and recognition [24,25]. Linking this framework to evidence from the UAE and wider healthcare literature: The rise in occupational stress and burnout among HCWs in the UAE can be traced to elevated job demands, high patient loads, shift rotations, emotional labor during crises like COVID-19, work-life imbalance, and insufficient organizational support [2,8]. These factors fit neatly into the “job demands” component of the JD-R model and have been consistently linked to exhaustion and lower well-being [5,14,21]. When job resources are lacking, such as reduced recognition, limited autonomy, or poor leadership, workers are more likely to experience burnout, characterized by emotional exhaustion and disengagement [9,26]. The JD-R theory posits two primary processes: a health-impairment process (where excessive job demands drain energy and cause burnout or physical/mental health issues), and a motivational process (where adequate resources foster work engagement, satisfaction, and retention) [21,24]. Empirical results in healthcare show that social support, positive leadership, and opportunities for professional growth (key resources) buffer the impacts of demand and reduce burnout risk [8,11,12].

During the COVID-19 pandemic, the JD-R framework became especially salient: Demands increased dramatically (e.g., greater emotional demands, fear of infection, unpredictable shifts), while resources such as personal protective equipment, managerial support, and clear communication did not always keep pace [8–10]. Studies from the UAE and similar regions illustrate a resulting spike in dissatisfaction, exit intentions, mental health complaints, and burnout [14,15,17].

The observed interplay of high job demands (workload, emotional strain, shift work) and insufficient job resources (support, recognition, opportunity) among UAE healthcare workers closely aligns with the dual processes articulated in the JD-R theory. Interventions aimed at reducing burnout and improving satisfaction, such as promoting support, recognition, autonomy, and career pathways, are consistent with JD-R-based recommendations from international research. These findings underscore the crucial need to address both sides of the equation: not only mitigating excessive demands but also enhancing resources to sustain health, satisfaction, and retention in the healthcare workforce.

Despite the growing body of international and regional literature, significant knowledge gaps remain regarding the predictors and interplay of occupational stress, job satisfaction, and burnout in the UAE’s diverse healthcare environment. While previous studies in the UAE have provided valuable insights, they have often been limited by single-center designs [7,9] or relatively small sample sizes [8], which can restrict the generalizability of the findings. Furthermore, a comprehensive assessment that concurrently employs validated scales to measure occupational stress, job satisfaction, and burnout within the JD-R framework is lacking. The originality and added value of this study are therefore threefold. First, it utilizes a relatively large sample size (N = 498) enhancing the statistical power and reliability of the analysis. Second, it is, to our knowledge, one of the first multicenter studies on this topic in the UAE, recruiting participants from various healthcare facilities, which improves the representativeness of the results. Finally, it provides a holistic investigation by integrating the use of validated scales for all key variables within a strong theoretical framework (the JD-R model), allowing for a subtle analysis of their interrelationships that has not been previously reported in this specific context. Identifying these predictors is crucial for informing leadership, implementing targeted interventions, and ultimately ensuring the sustainability of the healthcare workforce in the UAE [8,12]. Therefore, the current study aimed to investigate the influence of occupational stress and job satisfaction on burnout and to identify the key predictors of burnout among HCWs in the UAE.

## Methods

### Research design

The study employed a cross-sectional design. The participants were recruited from governmental hospitals and primary healthcare centers managed by Emirates Health Services (EHS). A cross-sectional design was employed in this study, as it enables the assessment of occupational stress, job satisfaction, and burnout among healthcare workers at a single point in time. This approach is effective for identifying prevalence, examining associations, and exploring potential predictors without requiring long-term follow-up. Given the study’s aim to understand current workplace conditions and their impact on healthcare workers, a cross-sectional design offered a practical and efficient method to gather relevant data across a large sample within a real-world healthcare setting.

### Sampling procedure and sample size estimation

The target population in this study consisted of healthcare workers (HCWs), while the accessible population comprised HCWs working in hospitals and primary healthcare centers under the EHS. Participants were included in this study if they had two years or more of experience, could speak and understand English, and were willing to participate voluntarily. This study employed a convenience sampling technique to recruit healthcare workers (HCWs) who voluntarily agreed to participate in the study. The sample size was calculated *a priori* using G*Power software version 3.1.9.7, based on the statistical tests required to address the research questions. For a correlation test (two independent Pearson’s r) with a hypothesized moderate correlation of *r = 0.3* between the variables, a minimum of 474 HCWs was required to achieve a statistical power of 0.90 at a two-tailed significance level of *α = 0.05*. A total of 521 HCWs agreed to participate, of which 498 completed the survey, yielding a response rate of 95.5%, which exceeded the minimum sample size requirement.

### Data collection procedure

Data was collected between 17 September and 30 November 2022 using an online survey that combined the study’s measuring instruments. The online survey was communicated to the research departments at EHS and distributed to all HCWs, inviting them to participate in the study. Participants were asked to give their written consent before participation. The survey was distributed over a two-month period, and the EHS research officer sent a weekly reminder. All responses were received by the Principal Investigator and saved in a secure password-protected device.

### Data collection tools

Demographic Data Questionnaire: A demographic data questionnaire was developed for this study, asking participants for general socio-demographic information.

The Work Stress Questionnaire (WSQ): Occupational stress was measured using the Work Stress Questionnaire (WSQ). The questionnaire contains 21 items covering four primary subscales: indistinct organization and conflicts, individual demands and commitment, influence at work, and work-to-leisure time interference. Participants rated their responses to each question on a four-point Likert scale, ranging from 1 (“Yes, always”) to 4 (“No, never”). The questionnaire was tested for reliability among industrial workers and found to be reliable with a Cronbach’s alpha of 0.934 [27]. The internal consistency of the WSQ was acceptable (Cronbach’s α = 0.879; McDonald’s ω = 0.843). Two scoring protocols were used for the Work Stress Questionnaire (WSQ): continuous and categorical. Continuous scores were calculated for each of the four subscales (Influence at Work, Indistinct Organization and Conflicts, Individual Demands, and Leisure Time) by summing responses to their respective items, with higher scores indicating greater stress in each domain. For categorical classification, participants were classified into low, moderate, and high stress levels for each subscale separately. As population-based normative cut-offs for the WSQ subscales are not established, thresholds were determined using the distribution of scores within our sample, a method commonly employed for deriving clinical categories in the absence of validated norms [28,29]. The specific cut-off points were based on tertiles (33rd and 66th percentiles) for each subscale as follows: Influence at Work (Low (≤7), Moderate (8 –9), High (≥10)), Indistinct Organization & Conflicts (Low (≤3), Moderate (4 –8), High (≥9)), Individual Demands (Low (≤10), Moderate (11 –17), High (≥18)), Leisure Time (Low (≤6), Moderate (7), High (≥8)) [30,31].

Maslach Burnout Inventory (MBI): Burnout was measured using the Maslach Burnout Inventory. The MBI contains 22 items and assesses three components of burnout: emotional exhaustion (9 items), depersonalization (5 items), and personal accomplishment (8 items). All items are scored using a 7-level frequency scale rated from 0 (Never) to 6 (Every day). The MBI subscales showed high internal consistency with Cronbach’s α coefficient values of 0.837, 0.869, and 0.88, respectively [32]. The questionnaire demonstrated satisfactory internal consistency reliability in this study, with Cronbach’s alpha of 0.860 and McDonald’s omega of 0.849. Two scoring protocols were used in this study: continuous and categorical. The continuous scoring protocol involved computing the average scores of individual items within each of the MBI’s three dimensions: emotional exhaustion, depersonalization, and personal accomplishment. Higher scores on the emotional exhaustion and depersonalization subscales indicated greater levels of emotional exhaustion and depersonalization, respectively, reflecting higher degrees of burnout. Higher personal accomplishment scores indicate lower levels of burnout in this domain. In contrast, based on established cut-off, the categorical scoring protocol classified participants into predefined degrees of burnout (low, moderate, high) for each dimension [33,34].

The Minnesota Satisfaction Questionnaire (MSQ-Short Version): Job satisfaction was measured using the Minnesota Satisfaction Questionnaire (MSQ) [35]. The short version of the MSQ comprises 20 items, each rated on a 5-point Likert scale, ranging from 1 (not satisfied) to 5 (extremely satisfied). The MSQ short version demonstrated satisfactory test-retest reliability with a Cronbach α coefficient of 0.89. In this study, the internal consistency of the MSQ was acceptable (Cronbach’s α = 0.948; McDonald’s ω = 0.947). Responses were computed according to the MSQ scoring protocol by calculating the mean scores for each subscale, with higher mean scores indicating higher job satisfaction.

### Ethical considerations

This study received dual ethical approvals from the Research Ethics and Integrity Committee (REIC) at the Higher Colleges of Technology (HCT). Abu Dhabi, UAE, on September 27, 2022 (REIC-Sep-2022–4), and from the Research Ethics Committee at the Ministry of Health and Prevention on October 20, 2022 (MOHAP/DXB-REC/ SOO/No. 95/ 2022). Participation was anonymous and subject to withdrawal at any time without any reason. Participants who agreed to enroll were asked to sign an informed consent.

### Data analysis

Coding for variables was performed following the scoring protocols for each measurement instrument. The data were analyzed using IBM SPSS Statistics (Version 29, 2022). Descriptive statistics were reported in mean and standard deviation, and categorical variables were reported in frequency and percentages. Pearson’s product-moment correlation coefficient test examined the associations between occupational stress, burnout, and job satisfaction. A stepwise multiple regression was used to predict the best-fit model and the independent predictors for the burnout domains. Prior to conducting correlation and regression analyses, the assumptions were examined. To avoid redundancy, either total scores or subscale scores were entered into the regression models, but not both simultaneously. Regression assumptions, including linearity, normality, homoscedasticity, independence of residuals, and multicollinearity, were examined prior to analysis. Multicollinearity was assessed through the Variance Inflation Factor (VIF) and tolerance values, all of which were within acceptable limits (VIF < 5, tolerance > 0.50), confirming that multicollinearity was not a concern. All assumptions were met, supporting the validity of the correlation and regression analyses. Demographics and other participants’ characteristics, as well as total scores and domains of occupational stress and job satisfaction, were entered into this analysis. To meet the assumption of stepwise regression, several demographic variables were re-coded into binomial categories. Specifically, income was dichotomized as >10,000 AED vs. ≤ 10,000 AED, marital status as married vs. unmarried (single, divorced, widowed), employment status as full-time vs. part-time, education level as Master’s/PhD vs. college/bachelor’s degree, nationality as Emirati vs. non-Emirati, and weekly working hours as >40 vs. ≤ 40 hours. These recoding decisions were based on the distribution of the data, the need to ensure adequate cell sizes for regression analysis, and consistency with prior research on occupational health and burnout. All entered variables in stepwise regression were either continuous or binomial, so some variables were recorded as binomial. A P-value ≤ 0.05 was considered statistically significant in all the analyses.

## Results

Table 1 presents the sociodemographic and occupational characteristics of the 498 healthcare workers enrolled in the study. The majority were nurses (55%), female (77.5%), and married (69.9%), with an average age of 36.24 years (SD = 8.86). Most participants held a bachelor’s degree (62.9%), were employed full-time (90.4%), and had more than ten years of experience (51%). Regarding income, 45.8% reported earnings between 5,001 and 10,000 AED. Participants were primarily based in Fujairah for living (36.1%) or working (36.9%), which represented the highest proportion among all emirates, held non-managerial positions (77.3%), worked 41–50 hours per week (49.4%), and were mainly assigned to the outpatient department (16.3%) or medical-surgical wards (15.9%).

**Table 1:** Participants’ characteristics (N = 498).

Variable		n (%) or Mean ± SD
**Age**		36.24 ± 8.86
Gender	Male	112 (22.5)
	Female	386 (77.5)
Total		498 (100)
**Marital Status**	Single	126 (25.3)
	Married	348 (69.9)
	Divorced	12 (2.4)
	Widowed	7 (1.4)
	Separated	5 (1.0)
Total		498 (100)
**Nationality**	Emirati	147 (29.5)
	Gulf Region	11(2.2)
	Middle Eastern Countries	74 (14.9)
	South Asia	102 (20.5)
	North Africa Countries	21 (4.2)
	South Africa Countries	5 (1.0)
	European	2 (0.4)
	Canada or the USA	4 (0.8)
	Australian	2 (0.4)
	Others	130 (26.1)
**Total**		498 (100)
**Education**	Diploma Degree	67 (13.5)
	Bachelor’s Degree	313 (62.9)
	Master’s Degree	90 (18.1)
	Doctorate Degree	28 (5.6)
**Total**		498 (100)
**Employment status**	Part-time	49 (9.8)
	Full-time	449 (90.2)
**Total**		498 (100)
**Occupation**	Doctor	98 (19.7)
	Nurse	274 (55.0)
	Midwife	7 (1.4)
	Pharmacist	21 (4.2)
	Dentist	20 (4.0)
	Environmental/Occupational Health and Hygiene Professionals	3 (0.6)
	Dietician	7 (1.4)
	Optometrist	3 (0.6)
	Medical Imaging Technician	19 (3.8)
	Medical and Pathology Laboratory Technician	9 (1.8)
	Respiratory Therapist	3 (0.6)
	Quality Control	1 (0.2)
	Other	33 (6.6)
**Total**		498 (100)
**Employment area**	ICU	27 (5.4)
	NICU	17 (3.4)
	Cath Lab	15 (3.0)
	Radiology	16 (3.2)
	ER	37 (7.4)
	Labor and Delivery	13 (2.6)
	Pediatrics	32 (6.4)
	Outpatient Department	84 (16.9)
	Medical-Surgical	76 (15.3)
	Oncology	5 (1.0)
	Operating Theater	20 (4.0)
	Recovery/PACU	1 (0.2)
	Mental/Psychiatric	12 (2.4)
	Infectious	5 (1.0)
	Quality	1 (0.2)
	Other	137 (27.5)
**Total**		498 (100)
**Job level**	Managerial	113 (22.7)
	Non-managerial	385 (77.3)
**Total**		498 (100)
**Weekly working hours**	≤ 20	13 (2.6)
	21–30	39 (7.8)
	31–40	165 (33.1)
	41–50	246 (49.4)
	> 50	35 (7.0)
**Total**		498 (100)
**Experience, years**	< 1 year	38 (7.6)
	1–4 years	95 (19.1)
	5–7 years	48 (9.6)
	8–10 years	63 (12.7)
	> 10 years	254 (51.0)
**Total**		498 (100)
**Income**	0–5,000 AED	33 (6.6)
	5,001–10,000 AED	228 (45.8)
	10,001–15,000 AED	37 (7.4)
	15,001–20,000 AED	35 (7.0)
	20,001–25,000 AED	83 (16.7)
	More than 25,000 AED	82 (16.5)
**Total**		498 (100)
**City of residence**	Abu Dhabi	34 (6.8)
	Dubai	90 (18.1)
	Sharjah	127 (25.5)
	Fujairah	180 (36.1)
	Umm Al Quwain	10 (2.0)
	Ras Al Khaimah	17 (3.4)
	Ajman	33 (6.6)
	Al-Ain	7 (1.4)
**Total**		498 (100)
**City of employment**	Abu Dhabi	42 (8.4)
	Dubai	117 (23.5)
	Sharjah	109 (21.9)
	Fujairah	184 (36.9)
	Umm Al Quwain	12 (2.4)
	Ras Al Khaimah	16 (3.2)
	Ajman	12 (2.4)
	Al-Ain	6 (1.2)
**Total**		498 (100)

Participants’ total scores of occupational stress, job satisfaction, and burnout were reported in Table 2. The mean score for the total occupational stress was 34.68 (SD = 10.15). Among the occupational stress subscales, the highest mean score, adjusted for the number of items, was observed for the “work-to-leisure time interference” subscale (6.12 ± 2.25), followed by the “influence at work” subscale (7.79 ± 2.44).

**Table 2:** Participants’ average total scores of scales and subscales of occupational stress, job satisfaction, and burnout (N = 498).

Scales/Subscales	Mean ± SD
Occupational Stress (WSQ)-Total score	34.68 ± 10.15
Indistinct Organization and Conflicts	7.14 ± 6.46
Individual Demands and Commitment	13.63 ± 6.59
Influence at Work	7.79 ± 2.44
Work to Leisure Time Interference	6.12 ± 2.25
Job Satisfaction (MSQ-SF)-Total score	3.13 ± 0.75
Intrinsic Satisfaction	3.04 ± 0.75
Extrinsic Satisfaction	3.22 ± 0.81
General Satisfaction	3.11 ± 0.88
Burnout (MBI)-Total score	66.78 ± 16.86
Emotional Exhaustion	26.41 ± 12.67
Depersonalization	11.40 ± 7.02
Personal Accomplishment	28.97 ± 8.34

The mean job satisfaction score was 3.13 (SD = 0.75), with the extrinsic subdomain yielding the highest mean score (M = 3.22, SD = 0.81). Regarding burnout, the overall mean total burnout score was 66.79 (SD = 16.86), with the highest mean score among its subscales being for “personal accomplishment” (28.97 ± 8.34), followed by “emotional exhaustion” (26.41 ± 12.67).

Table 3 summarizes participants’ levels of occupational stress and burnout across different subscales. The classification of HCWs stress using sample tertiles revealed distinct distributions across the WSQ subscales. For occupational stress, Indistinct Organization & Conflicts recorded the highest percentage of moderate to high levels (n = 283, 56.8%), followed by Individual Demands (n = 332, 66.6%). In contrast, the majority of participants (n = 342, 68.7%) reported low levels of stress related to Leisure Time, indicating it was the least severely impacted domain. Influence at Work was also a significant concern, with over half of the sample (n = 273, 54.8%) experiencing moderate to high stress levels. For burnout, depersonalization recorded the highest percentage of moderate to high levels (76.3%), followed by emotional exhaustion (71.3%). In contrast, the majority of participants (67.9%) reported low levels of reduced personal accomplishment, indicating that this was the least affected dimension of burnout.

**Table 3:** Participants’ categories of occupational stress and burnout (N = 498).

Scales/Subscales	Low Degree n (%)	Moderate Degree n (%)	High Degree n (%)
Occupational Stress (WSQ)			
*Indistinct Organization and Conflicts	215 (43.2)	117 (23.5)	166 (33.3)
*Individual Demands and Commitment	166 (33.3)	178 (35.7)	154 (30.9)
*Influence at Work	225 (45.2)	170 (34.1)	103 (20.7)
*Work to Leisure Time Interference	342 (68.7)	47 (9.4)	109 (21.9)
**Scales/Subscales**	**Low Degree** **n (%)**	**Moderate Degree** **n (%)**	**High Degree** **n (%)**
Burnout (MBI)			
Emotional Exhaustion	143 (28.7)	149 (29.9)	206 (41.4)
Depersonalization	118 (23.7)	153 (30.7)	227 (45.6)
Personal Accomplishment	338 (67.9)	98 (19.7)	62 (12.4)

*Note: Categories derived from sample tertiles (33rd/66th percentiles).

Table 4 presents Pearson’s correlation matrix examining the bivariate relationships among all measured continuous scales and subscales, including occupational stress, job satisfaction, and burnout. Age was included as a continuous variable to explore its potential associations with these psychological constructs. Concerning the association within the scales, the highest correlation was reported between the occupational stress total score and the domain of individual demands and commitment (r = 0.712, p ≤ 0.01), followed by the correlation between the total scores of occupational stress and indistinct organization and conflicts (r = 0.704, p ≤ 0.01). Regarding job satisfaction, the highest correlation was reported between the total score of job satisfaction and the domain of general satisfaction (r = 0.922, p ≤ 0.01), followed by the correlation between the total score of satisfaction and extrinsic satisfaction (r = 0.7918, p ≤ 0.01). Finally, regarding burnout, there was a weak negative correlation between personal accomplishment and emotional stress (r = −0.107, p ≤ 0.05) and depersonalization (r = −0.109, p ≤ 0.05). Table 4 also shows the correlation between the scales. Pearson’s correlation analysis revealed that the total score of occupational stress positively correlated with all domains, and the total score of job satisfaction had the highest correlation with extrinsic satisfaction (r = 0.327, ≤ 0.01). Also, total scores of occupational stress were found to be positively correlated with emotional stress (r = 0.492, p ≤ 0.01) and depersonalization (r = 0.306, p ≤ 0.01). Personal accomplishment showed weak negative correlations with emotional exhaustion (r = −0.107, p ≤ 0.05) and depersonalization (r = −0.109, p ≤ 0.05). Furthermore, the total job satisfaction score was only significantly associated with the emotional stress domain of burnout (r = −0.400, p ≤ 0.01).

**Table 4:** Correlation matrix of occupational stress, job satisfaction, burnout, and age (N = 498).

	Age	OS-TS	IOC	IDC	IW	WLTI	JS-TS	IS	ES	GS	ES	Depersonalization	PA
**Age**	1												
**OS-TS**	−0.088	1											
**IOC**	−0.048	0.704**	1										
**IDC**	−0.075	0.712**	0.098*	1									
**IW**	−0.156**	0.479**	0.068	0.366**	1								
**WLTI**	0.129**	−0.116**	−0.057	−0.394**	−0.192**	1							
**JS-TS**	−0.005	0.318**	0.046	0.336**	0.485**	−0.209**	1						
**IS**	−0.029	0.261**	0.041	0.260**	0.436**	−0.176**	0.918**	1					
**ES**	0.014	0.327**	0.047	0.361**	0.463**	−0.220**	0.913**	0.770**	1				
**GS**	−0.002	0.285**	0.039	0.300**	0.437	−0.180**	0.922**	0.774**	0.747**	1			
**ES**	−0.163**	0.492**	0.126**	0.590**	0.485**	−0.400**	0.513**	0.454**	0.476**	0.482**	1		
**Depersonalization**	−0.109*	0.306**	0.482**	0.022	−0.023	−0.040	−0.015	0.000	−0.041	−0.001	0.039	1	
**PA**	−0.064	−0.111*	−0.098*	−0.073	−0.028	0.025	−0.059	−0.052	−0.044	−0.064	−0.107*	0.109*	1

OS-TS: occupational stress-total score, IOC: indistinct organization and conflicts, IDC: individual demands and commitment, IW: influence at work, WTLI: work to leisure time interference, JS-TS: job satisfaction-total score, IS: intrinsic satisfaction, ES: extrinsic satisfaction, GS: general satisfaction, ES: emotional exhaustion, PA: personal accomplishment.

*Statistically significant at (α ≤ 0.05), **statistically significant at (α ≤ 0.01).

Table 5 presents the results of the stepwise multiple linear regression analysis for the burnout domains. Prior to interpreting the regression coefficients, statistical assumptions were verified. Multicollinearity was assessed using Variance Inflation Factor (VIF) and tolerance statistics. For all three models, VIF values ranged from 1.004 to 1.405 and tolerance values from 0.712 to 0.996, well within acceptable limits (VIF < 5, tolerance > 0.2), indicating no concerning multicollinearity. Only the best-fit models are shown. For emotional exhaustion, six factors significantly predicted 53% of the variance (R^2^ = 0.53, Adjusted R^2^ = 0.52). Positive predictors included individual demands and commitment, job satisfaction, influence at work, higher income, and unmarried status, indicating that higher levels of these variables were associated with greater emotional exhaustion. Work-to-leisure time interference was a negative predictor, suggesting that higher interference was associated with lower emotional exhaustion.

**Table 5:** Regression models predict burnout domains (N = 498).

	B	SE	Beta	T	p-value	Tolerance	VIF
**Dependent Variable: Emotional Exhaustion**							
Constant	−6.409	2.898		−2.212	0.027^*^		
Individual Demands and Commitment	0.669	0.073	0.345	9.208	< 0.001^***^	0.728	1.373
Job Satisfaction-Total Score	4.472	0.641	0.265	6.973	< 0.001^***^	0.727	1.375
Influence at Work	1.012	0.200	0.193	5.062	< 0.001^***^	0.712	1.405
Work to Leisure Time Interference	−0.876	0.199	−0.155	−4.395	< 0.001^***^	0.831	1.203
Income (> 10,000 AED vs. ≤ 10,000 AED)	2.586	0.833	0.102	3.104	0.002^**^	0.984	1.016
Marital Status (Unmarried vs. Married)	2.586	0.917	0.092	2.821	0.005^**^	0.983	1.017
**Model summary**: F = 85.579, p < 0.001^***^, R^2 ^= 0.530, Adjusted R^2^ = 0.523.							
**Dependent Variable: Depersonalization**							
Constant	10.211	2.216		4.608	< 0.001^***^		
Indistinct Organization and Conflicts	0.516	0.044	0.473	11.708	< 0.001^***^	0.996	1.004
Employment (Full Time vs. Part Time)	−2.270	0.959	−0.098	−2.368	0.018^*^	0.983	1.017
Income (> 10,000 AED vs. ≤ 10,000 AED)	1.237	0.587	0.087	2.110	0.035^*^	0.983	1.017
**Model summary**: F = 52.126 p < 0.001^***^, R^2 ^= 0.254, Adjusted R^2^ = 0.249.							
**Dependent Variable: Personal Accomplishment**							
Constant	33.511	2.848		11.766	< 0.001^***^		
Occupational Stress-Total Score	−0.120	0.040	−0.136	−2.962	0.003**	0.965	1.036
Education (Master’s & PhD vs. College and bachelor’s degree)	3.247	0.977	0.156	3.324	0.001^***^	0.865	1.156
Age	−0.160	0.051	−0.157	−3.129	0.002^**^	0.864	1.158
Nationality (Non-Emirati vs. Emirati)	2.612	0.967	0.134	2.702	0.007^**^	0.799	1.251
Weekly Working Hours (> 40 vs. ≤ 40 hours)	−2.013	0.806	−0.114	−2.497	0.013^*^	0.992	1.008
**Model summary**: F = 6.767, p < 0.001^***^, R^2 ^= 0.069, Adjusted R^2^ = 0.059.							

* α ≤ 0.05, ** α ≤ 0.01, *** α ≤ 0.001.

For depersonalization, three factors predicted 25.4% of the variance (R^2^ = 0.254, Adjusted R^2^ = 0.253). Indistinct organization and conflicts, as well as higher income, were positively associated with depersonalization, whereas full-time employment was a negative predictor, indicating that full-time employees had lower depersonalization scores compared to part-time employees.

For personal accomplishment, five factors predicted 6.9% of the variance (R^2^ = 0.069, Adjusted R^2^ = 0.059). Positive predictors included higher education level (Master’s/PhD) and non-Emirati nationality, which were associated with higher personal accomplishment. Negative predictors were total occupational stress score, older age, and working more than 40 hours per week, indicating that higher stress, older age, and longer working hours were associated with lower personal accomplishment. These findings suggest that different dimensions of occupational stress and job satisfaction impact the various burnout domains, with some factors contributing to increased burnout outcomes and others to decreased burnout outcomes.

## Discussion

The findings of this study provide valuable insights into the potential relationships between occupational stress, job satisfaction, and burnout among healthcare workers in the UAE, as well as identify the predictors of burnout. The mean occupational stress score was 34.68 ± 10.15, which aligns with the literature indicating the level of stress healthcare workers face in their working environment, particularly during the COVID-19 pandemic. For example, the study by Ali Jaber and Waheed [36] Among Saudi Arabia’s frontline healthcare workers, a similar scenario was reported, where the increased number of patients and care demands during the COVID-19 pandemic have raised the level of occupational stress to a high degree. This is further supported by a study from Ukraine that pinpointed socio-psychological factors contributing to increased stress among medical personnel during the pandemic [37]. The results of subscales from this current study identified that “work-to-leisure time interference” and “influence at work” are the most problematic areas. This finding is in harmony with the work of Bektaş, Misirlioğlu [38], who reported that organizational factors significantly influence job satisfaction, including the balance between work and personal life. The interference of work demands with personal time has been associated with increased stress and reduced job satisfaction, a trend that has been shown in several previous studies conducted in healthcare settings [39].

The mean score of 3.13 reflects a moderate level of job satisfaction among participants, with the highest score in the “extrinsic” subscale. This finding aligns with research conducted in the Emirates, where job satisfaction among healthcare workers was significantly influenced by extrinsic factors such as salary and benefits [40]. The relationship between job satisfaction and burnout is particularly critical, as evidenced by the high emotional exhaustion score reported in this study. The inverse relationship between job satisfaction and burnout has been reported in various studies, indicating that low job satisfaction is often coupled with a high level of burnout among healthcare workers [41,42]. The current study’s results on burnout, particularly regarding the ‘personal accomplishment’ dimension, suggest that participants may feel a sense of accomplishment in their work.. However, despite this sense of accomplishment, emotional exhaustion emerged as the most prevalent symptom of burnout, suggesting that healthcare workers experience substantial emotional strain even while feeling professionally competent. This can be supported by the literature, which states that although health professionals feel fulfilled in their jobs, they report remarkable burnout conditions [43]. These findings may have significant implications, suggesting that targeted interventions could be beneficial for enhancing job satisfaction and mitigating burnout in high-stress hospital environments.

The distribution of occupational stress revealed distinct patterns across different domains. The most prevalent source of moderate-to-high stress was Indistinct Organization & Conflicts, affecting over half of the sample (56.8%). This was closely followed by Individual Demands (66.6% moderate-to-high). In contrast, the domain of Leisure Time was the least affected for the majority of healthcare workers (68.7% low stress), though it remained a significant issue for a substantial minority (21.9% high stress). This pattern aligns with the literature, highlighting that organizational issues and high personal demands, rather than just work-life balance, are endemic stressors within healthcare settings, a trend exacerbated during periods of increased workload such as the COVID-19 pandemic. For instance, Sethuraman’s comparative analysis reveals that healthcare professionals have had to endure unforeseen stress levels during and after the pandemic, necessitating targeted interventions to mitigate these effects.

The high levels of moderate and severe burnout, particularly in ‘depersonalization’ and ‘emotional exhaustion’, indicate potential ongoing mental health concerns among HCWs. These findings are consistent with studies in various Middle Eastern regions suggesting that burnout is a serious concern among health professionals. For example, and Alhawassi [42] identified that among Saudi Arabian health workers, the symptoms of burnout are characterized by emotional exhaustion and depersonalization; hence, there is a dire need for efficient coping strategies and organizational support. Similarly, Abatay’s [42] study on burnout levels during the pandemic also revealed higher levels of emotional exhaustion and depersonalization among health professionals in direct contact with patients. Thus, they supported the result of the present study. The association between occupational stress and burnout has been well-documented in the literature. Kabunga’s study highlights how dysfunctional coping mechanisms, such as avoidance and denial, are positively related to higher levels of burnout. Hence, measures taken while handling the stressor play a significant role in overall well-being [44]. This becomes particularly relevant when applied to the current study and may relate well to highly interfered conditions at work-leisure times that act in tandem to contribute to the emotional exhaustion and depersonalization observed among subjects. In any event, this highlights the paramount necessity of adopting adaptive coping strategies in the pursuit of social support and practicing self-care [44]. The findings of this study are also consistent with those of studies conducted in the Emirates, where healthcare professionals reported similar levels of burnout and stress. Al-Omari, Mutair [43] noted that healthcare providers in both Saudi Arabia and the UAE experience significant burnout, driven by factors such as excessive workload, emotional demands, and inadequate support. From a regional perspective, this again highlights the importance of place-specific interventions, considering the very specific challenges faced by healthcare workers in the Middle East. The stepwise multiple linear regression analysis results provide valuable insights into the predictors of burnout among health workers in the three domains of emotional exhaustion, depersonalization, and personal accomplishment. The model predicting emotional exhaustion is significant, explaining the impact of individual demands, commitment, influence at work, work-to-leisure time interference, job satisfaction, income, and marital status. This aligns with previous literature that emphasizes the impact of job demand and work-life balance on burnout in healthcare professionals. For example, the increased levels of anxiety and burnout from Tohumcu [45] findings are closely linked to organizational commitment and intentions to leave the profession. This may suggest that increased levels of emotional exhaustion during the COVID-19 pandemic are associated with high job demands and lower job satisfaction, as argued by Tohumcu [45]. This aligns with findings in Emirati hospitals, where reduced satisfaction amplifies fatigue and intent to quit, particularly when compounded by stressful work environments [9]. Notably, “Work to Leisure Time Interference” had a negative association, suggesting that those who report more negative spillover into their personal life experience greater exhaustion; this is a pattern seen widely in healthcare, where long hours and unpredictable demands disrupt recovery and personal life [8]. Perceived “Influence at Work” (β = 0.193, p < 0.001) also emerged as significant. While autonomy is generally protective, the positive coefficient might indicate that in high-demand contexts, greater responsibility for outcomes without commensurate resources can heighten emotional burden, a phenomenon recognized in recent burnout literature [2]. Income and marital status both contributed significantly, with higher earnings and unmarried status linked to more exhaustion. Higher income’s relationship may reflect a selection effect, where those in higher-responsibility, more demanding roles are also better compensated [10].

Indistinct organizations and conflicts are significant predictors of depersonalization, further emphasizing the organizational factors that contribute to burnout. Most studies indicate that unclear roles and responsibilities at healthcare workplaces result in increased stress and burnout among workers [44]. Supporting the view that unclear role structures and workplace conflict foster psychological detachment and cynicism. Such findings are noted in both UAE and international research, where organizational ambiguity is repeatedly cited as driving disengagement and depersonalized care [12]. Depersonalization may also be influenced by employment status, specifically whether it is full-time or part-time, suggesting that job security and workload may impact how healthcare workers experience and cope with stress. This is further consistent with findings by Jusic [46], who noted that organizational factors greatly affect levels of burnout among health professionals. Higher income again predicted more depersonalization, reinforcing the interplay between greater demands at higher pay levels and emotional distancing as a coping mechanism [3].

On the other hand, personal accomplishment was the domain with the lowest percentage of variance explained, indicating that this domain is less influenced by the factors examined in this study. This might mean that personal accomplishment is more resilient to identifying stressors and may reflect the intrinsic motivations and fulfillment that health workers derive from their job. Nevertheless, personal accomplishment, while generally protective against burnout, may also be negatively impacted by high levels of occupational stress [47]. Empirical evidence supporting the link between high stress and burnout underscores the need for evidence-based policies to foster healthier workplaces and improve staff retention. In the UAE, recognizing the predictors of burnout within the distinct socio-cultural and organizational milieu enhances the precision of interventions and resource allocation within healthcare settings [7,8]. Moreover, the effective mitigation of occupational stress may prevent the onset or escalation of burnout, thereby preserving the functional capacity of healthcare systems and maintaining workforce morale [12,18,22]. Moreover, comprehensive meta-analyses and systematic reviews affirm the strong association between occupational stress and burnout across diverse healthcare systems. These analyses report consistent positive correlations between workload, emotional demands, and the risk of burnout, with effect sizes moderated by healthcare settings and cultural contexts [17].

The results also highlight the role of income and marital status as significant predictors of emotional exhaustion. High-income respondents reported low emotional exhaustion, consistent with previous findings that financial stability buffers one against stress and burnout [39]. Moreover, marital status further points out that support systems may have an essential buffering effect on burnout, in that married individuals have a means of emotional outlet to diffuse their work-related stressors [48]. Age and longer working hours reduced accomplishment, echoing findings that prolonged exposure to stress and fatigue can undermine intrinsic reward and professional efficacy [5].

### Limitations

The findings from this study should be interpreted with several limitations in mind. First, the cross-sectional design allows only a limited establishment of a causal relationship between occupational stress, job satisfaction, and burnout. Second, reliance on self-reported measures may introduce response bias because participants overestimate or underestimate their experiences. In addition, the research was conducted within a specific cultural and organizational context in the UAE, where unique social norms, workplace hierarchies, and healthcare practices may influence experiences of stress, burnout, and job satisfaction. These contextual factors may limit the generalizability of the findings to other regions or healthcare systems with different cultural and organizational characteristics. Furthermore, the regression models explained only a modest proportion of the variance in some outcomes, particularly in the personal accomplishment domain, which suggests that other influential variables may not have been captured. The potential for multicollinearity among predictors and the absence of control for certain confounding variables may also limit the precision and generalizability of the predictive findings. Future research, employing longitudinal designs and encompassing diverse healthcare environments, is recommended to address these limitations and provide a more comprehensive understanding of these variables.

### Implications

These findings underscore the importance of health organizations implementing effective policies to mitigate burnout among healthcare workers. Since individual demands, job satisfaction, and work-to-leisure interference are major predictors of emotional exhaustion, organizations should implement workload management policies and promote work-life balance initiatives. Increased job satisfaction through recognition programs and professional development opportunities can protect against burnout. Moreover, depersonalization can be reduced by enhancing organizational clarity and providing adequate support systems. Additionally, tailored psychological well-being interventions for healthcare settings are recommended to support staff mental health further. Such implications suggest targeted interventions that will improve not only employees’ well-being but also the quality of care provided to patients, ultimately benefiting the entire healthcare system.

## Conclusion

The present study examined the interrelationships among occupational stress, job satisfaction, and burnout among healthcare workers, highlighting the critical factors that contribute to emotional exhaustion, depersonalization, and personal accomplishment. The findings indicate that occupational stressors, particularly individual demands and commitment, significantly predict emotional exhaustion, accounting for a substantial portion of the variance in this domain. Notably, job satisfaction emerged as a key protective factor, mitigating the impact of stress on emotional exhaustion, which underscores the value of fostering a supportive and motivating work environment for healthcare professionals.

The results further show that indistinct organizational structures and conflicts significantly predict depersonalization, while demographic factors, including income and employment status, influence both emotional exhaustion and depersonalization. The relatively low variance in personal accomplishment suggests that this domain is more resilient to occupational stress, with intrinsic motivation and personal fulfillment acting as potential buffers against burnout.

Overall, the study emphasizes the imperative need for targeted organizational interventions, including workload management policies, promotion of work-life balance, organizational clarity, recognition programs, and tailored psychological well-being initiatives. Such strategies can reduce burnout, enhance job satisfaction, and support the mental health and resilience of healthcare workers, ultimately improving the quality of care provided to patients. Future research should examine these relationships across diverse healthcare settings to develop comprehensive, context-specific strategies for sustaining a healthy and motivated workforce.

In the context of the UAE, these recommendations should be operationalized by aligning with ongoing national healthcare reforms, such as accreditation-driven quality improvement initiatives, and investing in employee wellness programs in both public and private hospitals. Strategies should consider the multicultural composition of the workforce, existing staff shortages, and institutional efforts to standardize work conditions across emirates. Tailoring interventions to these systemic realities would enhance their feasibility, impact, and contribution to health policy in the UAE.

## Abbreviations

HCWs: Healthcare Workers
UAE: United Arab Emirates
WSQ: Work Stress Questionnaire
MBI: Maslach Burnout Inventory
MSQ: Minnesota Satisfaction Questionnaire
